# Nomogram Model for Prognosis of Distant Metastatic DTC Based on Inflammatory and Clinicopathological Factors

**DOI:** 10.1210/jendso/bvaf037

**Published:** 2025-02-27

**Authors:** Chenghui Lu, Guoqiang Wang, Zengmei Si, Fengqi Li, Xinfeng Liu, Na Han, Congcong Wang, Jiao Li, Xufu Wang

**Affiliations:** Department of Nuclear Medicine, The Affiliated Hospital of Qingdao University, Qingdao 266003, PR China; Department of Nuclear Medicine, The Affiliated Hospital of Qingdao University, Qingdao 266003, PR China; Department of Nuclear Medicine, The Affiliated Hospital of Qingdao University, Qingdao 266003, PR China; Department of Nuclear Medicine, The Affiliated Hospital of Qingdao University, Qingdao 266003, PR China; Department of Nuclear Medicine, The Affiliated Hospital of Qingdao University, Qingdao 266003, PR China; Department of Nuclear Medicine, The Affiliated Hospital of Qingdao University, Qingdao 266003, PR China; Department of Nuclear Medicine, The Affiliated Hospital of Qingdao University, Qingdao 266003, PR China; Department of Nuclear Medicine, The Affiliated Hospital of Qingdao University, Qingdao 266003, PR China; Department of Nuclear Medicine, The Affiliated Hospital of Qingdao University, Qingdao 266003, PR China

**Keywords:** thyroid cancer, distant metastasis, inflammatory marker, prognosis, nomogram

## Abstract

**Context:**

Inflammatory markers may serve as potential biomarkers in predicting prognosis in patients with distant metastasis differentiated thyroid cancer (DM-DTC).

**Objective:**

This study aimed to evaluate the predictive ability of inflammatory markers and clinicopathological features for disease progression (PD) in patients with DM-DTC.

**Methods:**

A retrospective analysis was conducted on 230 DM-DTC patients from May 2016 to January 2022. Patients were divided into a training set and a validation set at a 7:3 ratio. Inflammatory markers were obtained within 1 week before the last ^131^I treatment. The primary outcome was progression-free survival (PFS). Univariable and multivariable Cox proportional hazards models identified significant prognostic factors, and a nomogram based on inflammatory markers and clinicopathological features was constructed and validated using R software.

**Results:**

Multivariable Cox regression analysis showed that age (hazard ratio [HR] = 2.191; 95% CI, 1.387-3.462), histological type (HR = 2.030; 95% CI, 1.216-3.389), distant metastatic site (HR = 3.379; 95% CI, 1.832-6.233), T stage (HR = 6.061; 95% CI, 2.469-14.925), and LMR (HR = 2.050; 95% CI, 1.194-3.519) were identified as independent risk factors for the progression of DM-DTC. A predictive nomogram was constructed to estimate the probability of DM-DTC progression. The C-index of the PFS model was calculated to be 0.775 (0.749-0.802) for the training set and 0.731 (95% CI, 0.686-0.775) for the validation set. The calibration curve of the validation set closely approached the reference line. The decision curve analysis indicated that when the risk threshold was greater than 0.2, this nomogram model provided clinical net benefit.

**Conclusion:**

The study identified significant inflammatory markers and clinical factors for predicting PD in DM-DTC patients, providing a robust prognostic model with potential clinical application.

Thyroid cancer stands as one of the most prevalent malignancies within the endocrine system, exhibiting a rising trend in incidence across the globe. Differentiated thyroid cancer (DTC) represents the majority of thyroid malignancies, constituting more than 90% of cases. While the majority of DTC patients enjoy a favorable prognosis, a significant subset, approximately 10%, present with distant metastasis at the time of diagnosis or develop distant metastasis during follow-up [1]. Among this subgroup, 25% to 50% are identified with radioiodine-refractory DTC (RAIR-DTC), a particularly aggressive phenotype characterized by a 5-year survival rate of less than 50% and a 10-year survival rate of less than 10% [2, 3]. Consequently, the accurate prognostication of disease progression and overall patient outcomes remains a complex and challenging endeavor. In the clinical setting, prognostic assessment tools are predominantly anchored in the clinicopathological attributes of patients, including but not limited to age, sex, tumor dimensions, lymph node involvement, histological type, and serum thyroglobulin (Tg) levels [4, 5]. Despite their utility, these parameters possess inherent limitations in predictive accuracy, underscoring the necessity for the integration of novel biomarkers and predictive algorithms to refine the precision and reliability of prognostic evaluations.

Inflammation's role as a pivotal catalyst in the advancement of cancer has garnered increasing recognition. A spectrum of systemic inflammatory biomarkers—such as the neutrophil-to-lymphocyte ratio (NLR), platelet-to-lymphocyte ratio (PLR), lymphocyte-to-monocyte ratio (LMR), and systemic immune-inflammation index (SII)—has emerged as promising prognostic indicators across a variety of malignancies, including DTC [6, 7]. These markers, easily accessible through standard blood tests, serve as a reflection of the equilibrium between protumorigenic inflammatory processes and antitumoral immune responses. Empirical evidence from prior research has underscored a correlation between elevated NLR and an adverse prognosis in a spectrum of cancers, notably advanced papillary thyroid carcinoma (PTC) [6]. The prognostic significance of PLR and LMR in DTC has also been scrutinized, yielding mixed results [7]. Despite this, the prognostic utility of these inflammatory biomarkers, particularly in forecasting the clinical outcomes of patients with distant metastasis DTC (DM-DTC), remains an open question.

The novelty of this study lay in the integration of peripheral blood inflammatory biomarkers into the prognostic evaluation of DM-DTC. We aimed to establish and validate a prognostic model that synergizes these biomarkers with traditional clinicopathological characteristics. This approach was envisioned to pave the way for a more efficacious, personalized management strategy tailored to the therapeutic needs of DM-DTC patients.

## Materials and Methods

### Study Patients

This retrospective analysis examined the peripheral blood parameters and clinical pathological data of 230 patients with DM-DTC who received ^131^I therapy at the Affiliated Hospital of Qingdao University between May 2016 and December 2021.

Inclusion criteria included the following: 1) previous total thyroidectomy with histopathological confirmation of DTC (PTC or follicular thyroid cancer [FTC]) and evidence of distant metastasis, either confirmed by pathology or suggested by imaging; 2) at least one course of ^131^I treatment and thyrotropin (TSH) less than 0.1 mU/L during TSH suppression treatment; and 3) Tg antibodies (TgAb) less than 115 IU/mL.

Exclusion criteria included the following: 1) history of infectious diseases or recent use of antibiotics, antiplatelet agents, hormones, or immunosuppressive drugs prior to the last ^131^I treatment; 2) concurrent malignancies or autoimmune or hematological disorders; and 3) combined with medullary thyroid carcinoma or anaplastic thyroid carcinoma or poorly DTC. The enrolled cases were divided into a training set and a validation set at a ratio of 7:3.

The studies involving human participants were reviewed and approved by the ethical committee of the Affiliated Hospital of Qingdao University (QYFY WZLL 27846). Written informed consent to participate in this study was provided by the participants.

### Data Collection

Clinical pathological data of the study participants, including age, sex, histological type, vascular cancer thrombus, T stage, N stage, sites of distant metastasis, and preablative stimulated thyroglobulin (sTg) levels, were collected through the electronic medical record system. The eighth edition of the American Joint Committee on Cancer for TNM staging was employed. The Tg level was measured by an electrochemiluminescence immunoassay assay (Roche Diagnostics; laboratory detection range of 0.04-500.00 ng/mL). When Tg was greater than 500 ng/mL, equal proportion dilution was performed. Complete blood counts were obtained within 1 week prior to the last ^131^I treatment.

### Calculation of Inflammatory Indicators

The NLR is the ratio of neutrophils to lymphocytes, PLR is the ratio of platelets to lymphocytes, and LMR is the ratio of lymphocytes to monocytes. The SII is calculated by multiplying the neutrophil and platelet counts and then dividing by the lymphocyte count.

### Treatment and Follow-Up

Patients discontinued levothyroxine treatment and followed a low-iodine diet for 2 to 4 weeks before ^131^I treatment. Routine evaluations, including serum Tg, TgAb, and TSH, complete blood counts, and neck ultrasound were performed before each course of ^131^I treatment. Additional imaging studies, such as computed tomography (CT), magnetic resonance imaging, whole-body bone scintigraphy, or ^18^F-FDG PET/CT were performed if there were suspicions of lung, bone, brain, or other distant metastases. Empirical treatment was administered with a single dose of 3700 to 7400 MBq per treatment. Repeated ^131^I treatment was administered 6 to 12 months following the latest treatment, provided that the lesions demonstrated satisfactory ^131^I uptake and an advantageous clinical response was observed. Serum TSH, Tg, and TgAb, as well as neck ultrasound and CT, were reevaluated every 3 to 6 months to assess the progression of disease. ^131^I treatment was discontinued in the event of disease progression, regardless of iodine uptake. The last follow-up was conducted in March 2024, and the end point for survival analysis was progression-free survival (PFS), defined as the time from the start of surgical treatment to progression of disease (PD) before treatment with other modalities after the discontinuation of radioiodine treatments. PD was evaluated by CT based on Response Evaluation Criteria in Solid Tumors (RECIST) 1.1 criteria. Patients who remained alive and had no evidence of PD at the last follow-up were censored.

### Statistical Analysis

Receiver operating characteristic (ROC) curves were used to determine the optimal cutoff values for SII, NLR, PLR, LMR, age, and sTg in predicting PD. Univariable and multivariable Cox proportional hazards models were used to analyze the prognostic factors. The statistical significance level was set at α = .05. Establishment and validation of the nomogram was performed, evaluating discrimination, calibration, and clinical usability. Harrell's concordance index (C-index) was calculated to assess the model's discrimination. Time-dependent ROC curves were plotted to evaluate the model's discriminatory ability. Calibration curves were generated by comparing predicted probabilities with observed probabilities at different time points to assess the model's calibration. Decision curve analysis was performed to evaluate the clinical usability and net benefit at different threshold probabilities. Statistical analysis and plotting were conducted using R software (version 4.1.3).

## Results

### Baseline Characteristics of Patients

**Figure 1.**
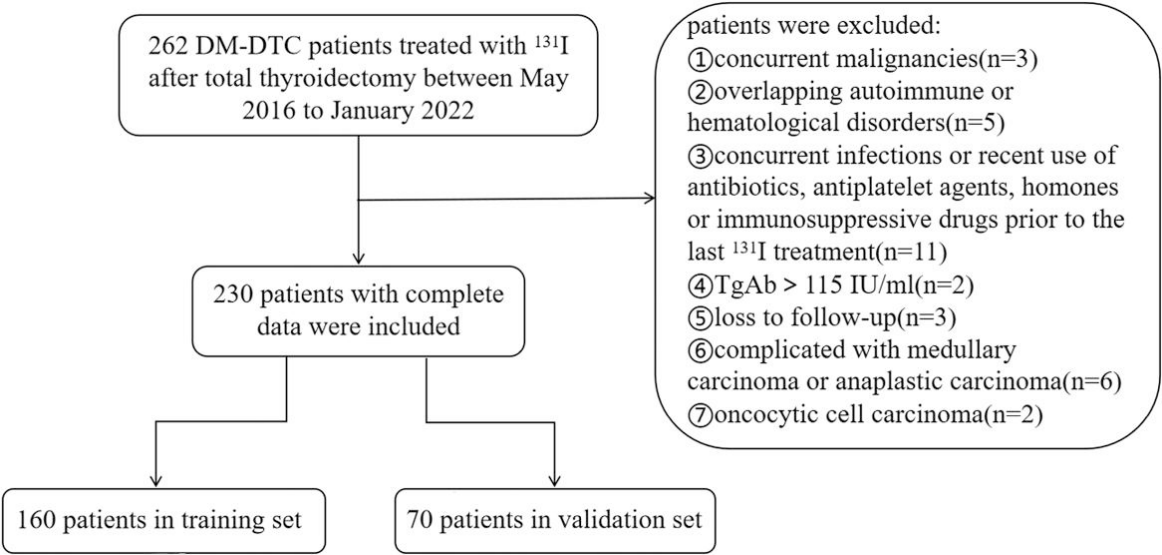
The flowchart of the enrolled patients.

The flowchart of patient enrollment is shown in Fig. 1. A total of 230 patients were included in this study, randomly divided into a training set (n = 160) and a validation set (n = 70) at a ratio of 7:3. The patients underwent 2 to 5 treatment courses, accumulating a total dose of 7400 to 27 750 MBq. At the end of the follow-up, 140 patients were identified as RAIR-DTC with 112 exhibiting PD and 28 maintaining stable disease. In total, there were 118 cases presented with PD while there were 112 with remission or stabilization. The median follow-up time for the training set and the validation set was 65 (38-84) months and 67 (36-82) months, respectively. The median PFS was 63 (29-78) months for the training set and 64 (26-80) months for the validation set.

**Figure 2.**
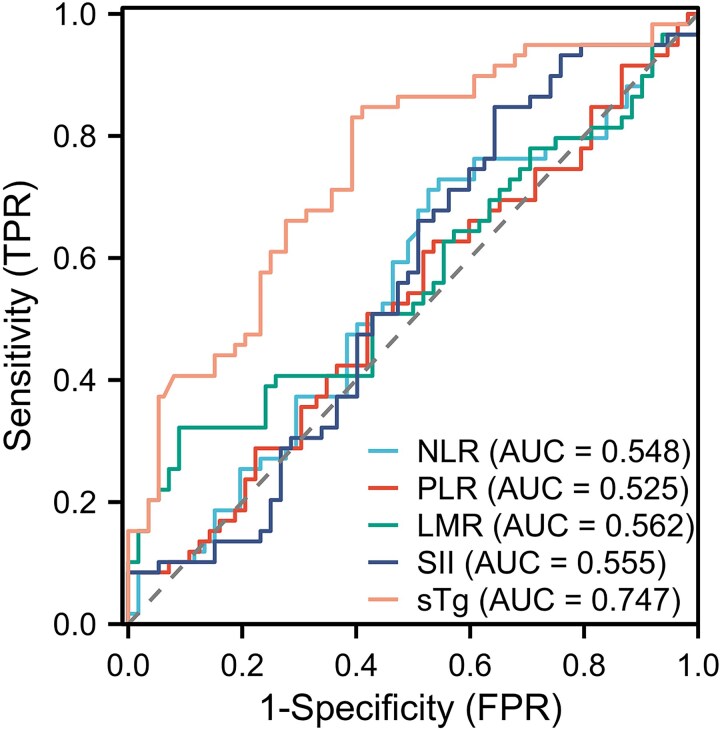
Receiver operating characteristic curves for the prediction of disease progression in distant metastasis differentiated thyroid cancer patients by various continuous variables.

ROC curve analysis showed that the area under the curve for NLR, PLR, LMR, SII, and sTg levels in predicting PD of DM-DTC patients before the last ^131^I treatment were 0.548 (95% CI, 0.473-0.623), 0.525 (95% CI, 0.450-0.599), 0.562 (95% CI, 0.487-0.637), 0.555 (95% CI, 0.479-0.631), and 0.747 (95% CI, 0.684-0.810), respectively, as shown in Fig. 2. The corresponding Youden index and optimal cutoff values are shown in Table 1. Patients were grouped based on the optimal cutoff values of SII, NLR, PLR, LMR, age, and sTg. The baseline characteristics of patients in the training set and the validation set are shown in Table 2.

**Table 1.** Performance of various continuous variables in predicting disease progression in distant metastasis differentiated thyroid cancer patients

	Se	Sp	Youden index	Cutoff
NLR	0.712	0.473	0.185	1.59
PLR	0.610	0.482	0.092	123.20
LMR	0.322	0.910	0.233	4.63
SII	0.847	0.357	0.205	316.22
sTg	0.831	0.607	0.438	61.85(μg/mL)

Abbreviations: LMR, lymphocyte-to-monocyte ratio; NLR, neutrophil-to-lymphocyte ratio; PLR, platelet-to-lymphocyte ratio; Se, sensitivity; SII, systemic immune-inflammation index; Sp, specificity; sTg, stimulated thyroglobulin.

**Table 2.** Baseline characteristics of distant metastasis differentiated thyroid cancer patients in the training set and the validation set

Characteristics	Training set	Validation set	*P*
n	160	70	
Event, n (%)			.404
0	75 (46.9%)	37 (52.9%)	
1	85 (53.1%)	33 (47.1%)	
Time, median (IQR)	1890 (870-2310)	1920 (780-2400)	.744
Sex, n (%)			.783
Female	90 (56.2%)	38 (54.3%)	
Male	70 (43.8%)	32 (45.7%)	
Age, n (%), y			.960
<55	84 (52.5%)	37 (52.9%)	
≥55	76 (47.5%)	33 (47.1%)	
Histological type, n (%)			.571
PTC	125 (78.1%)	57 (81.4%)	
FTC	35 (21.9%)	13 (18.6%)	
Vascular cancer thrombus, n (%)			.791
No	52 (32.5%)	24 (34.3%)	
Yes	108 (67.5%)	46 (65.7%)	
Distant metastatic site, n (%)			.355
Lung	120 (75%)	57 (81.4%)	
Bone	18 (11.2%)	8 (11.4%)	
Lung + bone	22 (13.8%)	5 (7.1%)	
T, n (%)			.908
T1 + T2	40 (25%)	17 (24.3%)	
T3 + T4	120 (75%)	53 (75.7%)	
N, n (%)			.878
N0 + N1a	45 (28.1%)	19 (27.1%)	
N1b	115 (71.9%)	51 (72.9%)	
SII, n (%)			.749
<316.22	38 (23.8%)	18 (25.7%)	
≥316.22	122 (76.2%)	52 (74.3%)	
NLR, n (%)			.411
<1.59	116 (72.5%)	47 (67.1%)	
≥1.59	44 (27.5%)	23 (32.9%)	
PLR, n (%)			.081
<132.2	42 (26.2%)	11 (15.7%)	
≥132.2	118 (73.8%)	59 (84.3%)	
LMR, n (%)			.624
≥4.63	128 (80%)	54 (77.1%)	
<4.63	32 (20%)	16 (22.9%)	
Tg, n (%)			.559
<61.85	66 (41.2%)	26 (37.1%)	
≥61.85	94 (58.8%)	44 (62.9%)	
Number of ^131^I treatments (n), median (IQR)	2 (2, 3)	3 (2, 3)	.385
Cumulative dose of ^131^I (mCi), median (IQR)	360 (300, 550)	435 (281, 600)	.302

Abbreviations: FTC, follicular thyroid cancer; IQR, interquartile range; LMR, lymphocyte-to-monocyte ratio; NLR, neutrophil-to-lymphocyte ratio; PLR, platelet-to-lymphocyte ratio; PTC, papillary thyroid carcinoma; SII, systemic immune-inflammation index; Tg, thyroglobulin.

### Univariable and Multivariable Cox Regression Analysis and Model Construction in the Training Set

**Figure 3.**
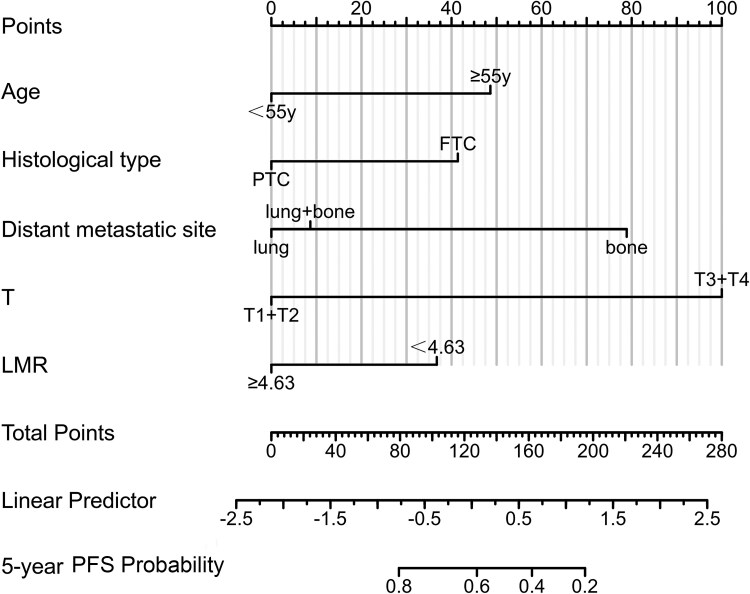
A nomogram predicting the risk of progression in distant metastasis differentiated thyroid cancer patients.

In the univariable COX regression analysis, variables that reached statistical significance (*P* < .1) were selected for inclusion in the subsequent multivariable Cox regression analysis. The findings revealed that age (hazard ratio [HR] = 2.191; 95% CI, 1.387-3.462), histological type (HR = 2.030; 95% CI, 1.216-3.389), distant metastatic site (HR = 3.379; 95% CI, 1.832-6.233), T stage (HR = 6.061; 95% CI, 2.469-14.925), and LMR (HR = 2.050; 95% CI, 1.194-3.519) were established as independent predictors of PD in DM-DTC (Table 3). Based on these 5 predictors, a predictive nomogram was constructed to estimate the probability of DM-DTC progression (Fig. 3). For instance, a case of a 60-year-old female postoperative patient with PTC, T2N1bM1, lung metastasis, and LMR less than 4.63 equates to a total score of 86 on the predictive nomogram. This score was prognostic, indicating a 5-year PFS probability close to 80%, thereby quantifying a favorable outcome in this patient's clinical trajectory.

**Table 3.** Univariate and multivariable Cox regression analysis of disease progression in distant metastasis differentiated thyroid cancer patients in the training set

Characteristics	Total, No.	Univariate analysis		Multivariable analysis	
		Hazard ratio (95% CI)	*P*	Hazard ratio (95% CI)	*P*
Sex	160				
Female	90	Reference			
Male	70	1.202 (0.781-1.850)	.402		
Age, y	160				
<55	84	Reference		Reference	
≥55	76	2.316 (1.493-3.593)	**<.001**	2.191 (1.387-3.462)	**<**.**001**
Histological type	160				
PTC	125	Reference		Reference	
FTC	35	3.221 (2.064-5.027)	**<**.**001**	2.030 (1.216-3.389)	.**007**
Vascular cancer thrombus	160				
No	52	Reference		Reference	
Yes	108	1.692 (1.047-2.740)	.**032**	0.796 (0.447-1.418)	.439
Distant metastatic site	160				
Lung	120	Reference		Reference	
Bone	18	3.891 (2.209-6.857)	**<**.**001**	3.379 (1.832-6.233)	**<**.**001**
Lung + bone	22	1.841 (1.051-3.227)	.**033**	0.933 (0.459-1.897)	.849
T	160				
T1 + T2	40	Reference		Reference	
T3 + T4	120	5.291 (2.439-11.494)	**<**.**001**	6.061 (2.469-14.925)	**<**.**001**
N	160				
N0 + N1a	45	Reference			
N1b	115	1.232 (0.756-2.008)	.402		
SII	160				
<316.22	38	Reference		Reference	
≥316.22	122	1.667 (0.948-2.933)	.076	0.784 (0.424-1.447)	.437
NLR	160				
<1.59	116	Reference			
≥1.59	44	0.948 (0.578-1.556)	.833		
PLR	160				
<132.2	42	Reference			
≥132.2	118	0.777 (0.486-1.242)	.292		
LMR	160				
≥4.63	128	Reference		Reference	
<4.63	32	2.375 (1.500-3.762)	**<**.**001**	2.050 (1.194-3.519)	.**009**
Tg	160				
<61.85	66	Reference		Reference	
≥61.85	94	3.230 (1.934-5.394)	**<**.**001**	1.463 (0.820-2.610)	.198
No. of ^131^I treatments	160	0.890 (0.723-1.096)	.273		
Cumulative dose of ^131^I	160	0.999 (0.998-1.001)	.279		

The values in bold indicates values with *P* < .05.

Abbreviations: FTC, follicular thyroid cancer; LMR, lymphocyte-to-monocyte ratio; NLR, neutrophil-to-lymphocyte ratio; PLR, platelet-to-lymphocyte ratio; PTC, papillary thyroid carcinoma; SII, systemic immune-inflammation index; Tg, thyroglobulin.

### Internal Validation of the Predictive Model

#### Discrimination validation

**Figure 4.**
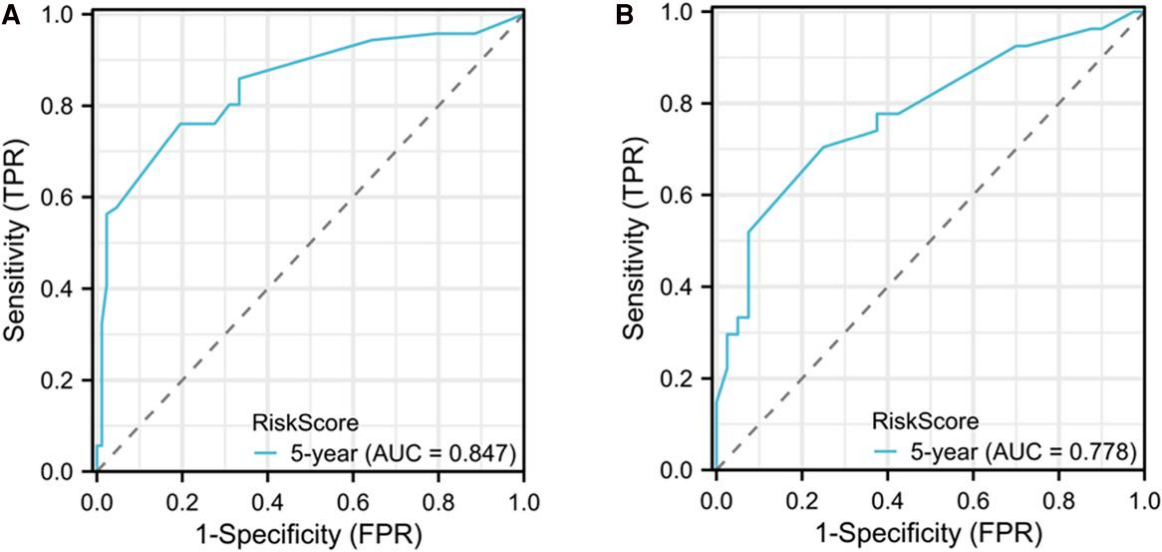
Receiver operating characteristic curve in a progression risk nomogram model for distant metastasis differentiated thyroid cancer patients. A, Ttraining set; B, validation set.

The C-index for the PFS model, derived from multivariable analysis, was determined to be 0.775 (0.749-0.802) for the training set and 0.731 (95% CI, 0.686-0.775) for the validation set. The time-dependent ROC curves for the 5-year PFS model were delineated for both the training and validation sets, as depicted in Fig. 4. The area under the curve for the training set at 5 years was 0.847 (95% CI, 0.7837-0.9098), while for the validation set, it was 0.778 (95% CI, 0.6616-0.8943).

#### Calibration validation

**Figure 5.**
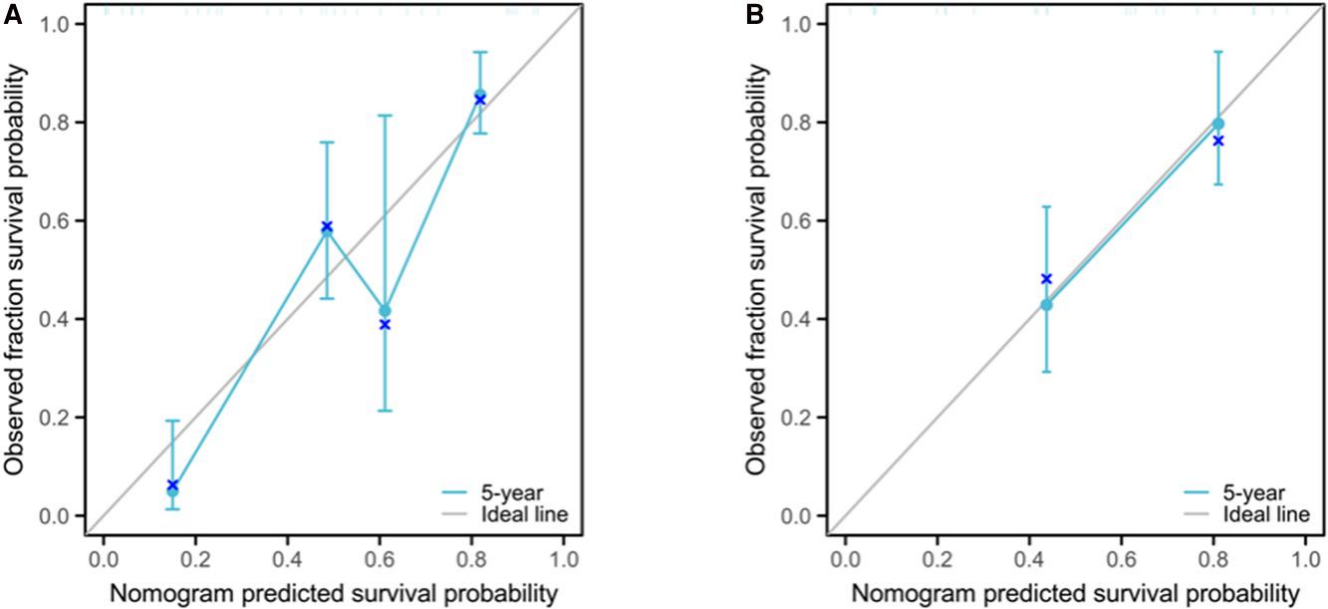
Calibration curve of a progression risk nomogram model for distant metastasis differentiated thyroid cancer patients. A, Training set; B, validation set.

The calibration of the prognostic model was meticulously assessed through the juxtaposition of the predicted probabilities with the empirical event rates at successive time intervals, graphically represented by the calibration curve. Calibration proficiency is denoted by the curve's proximity to the ideal reference line. Deviation from the diagonal line suggests a calibration discrepancy, indicative of the model's reduced accuracy in forecasting. The calibration curve for the 5-year PFS model is delineated in Fig. 5, illustrating a high degree of concordance between the predicted probabilities and the observed incidence of events across various time points in the validation cohorts.

### Clinical Decision Curve Analysis

**Figure 6.**
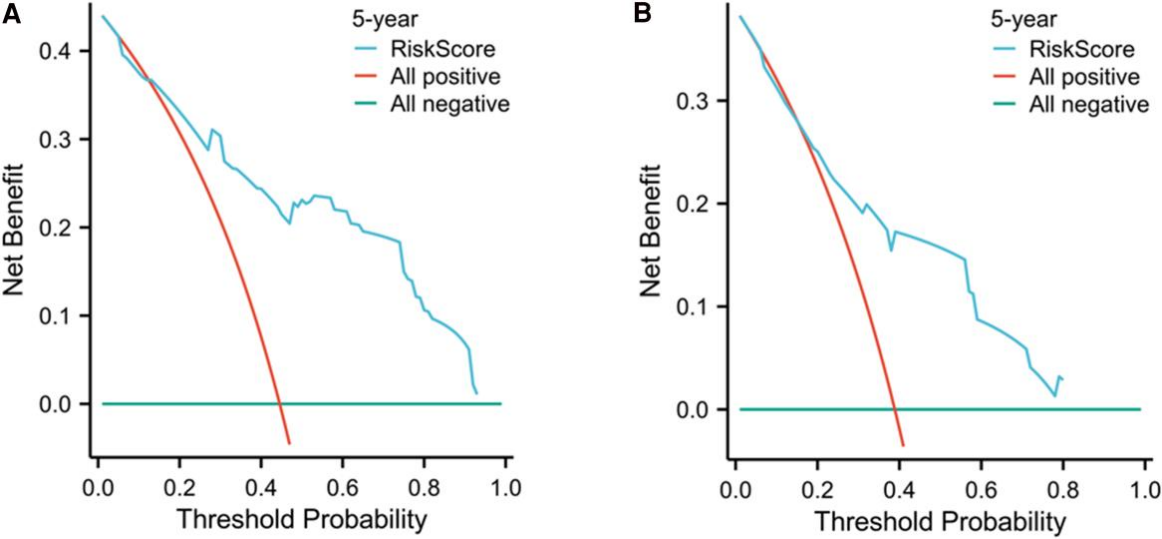
Clinical decision of a progression risk nomogram model for distant metastasis differentiated thyroid cancer patients. A, Training set; B, validation set.

The predicted probabilities for the 5-year outcome were calculated based on the model, and a clinical decision curve analysis was performed, as shown in Fig. 6. The x-axis represents the threshold probabilities based on the predicted model, and the y-axis represents the corresponding net benefit for patients at each threshold probability. In our study, the results of the decision curve analysis indicated that when the risk threshold was greater than 0.2, this nomogram model provided a clinical net benefit.

## Discussion

Inflammatory markers have emerged as potential prognostic indicators in various cancers, including DTC [8-10]. NLR, PLR, LMR, and SII are some of the markers that have been studied for their prognostic value. These markers reflect the systemic inflammatory response, which plays a critical role in cancer progression and metastasis. A recent meta-analysis concluded that while these markers are promising, more prospective data are needed to definitively establish their prognostic value in DTC [7]. However, previous studies mainly focused on the overall population of DTC patients, and their utility in predicting PD in DM-DTC patients has not been thoroughly investigated [11]. Our findings indicate that specific inflammation markers, such as the LMR, alongside clinical parameters like age, histological type, and N stage, are significant predictors of PD in DM-DTC. These results were consistent with those of previous studies [8-11], which have highlighted the prognostic value of inflammation markers in thyroid cancer progression. Furthermore, our findings underscored the efficacy of integrating these biomarkers into a nomogram for the predictive model of PD, which emphasizing the importance of a comprehensive model in clinical oncology for better prognostic accuracy.

An investigation leveraging the Surveillance, Epidemiology, and End Results (SEER) database has revealed that age 65 years and older, male, node-positive disease, and larger tumor dimensions are correlated with an elevated mortality risk in DM-DTC [12]. These findings were similar to those of our study, which showed that age and T stage were independent risk factors for predicting PD in DM-DTC. A substantial body of research has underscored the prognostic relevance of clinicopathological characteristics in thyroid cancer [13-16], establishing a widely recognized consensus.

Our findings indicated that histological type was one of the independent risk factors affecting the progression of DM-DTC. PTC is the most prevalent, accounting for approximately 80% of all thyroid cancers. It is characterized by a favorable prognosis and typically occurs in younger women. PTC often presents as microcarcinomas and has a propensity for lymphatic spread, although distant metastases are rare [17]. FTC comprises about 10% to 15% of thyroid malignancies, generally affecting older patients. FTC is more aggressive than PTC, with a higher likelihood of hematogenous spread, particularly to the lungs and bones. Its diagnosis often requires evidence of vascular invasion [18-20]. Furthermore, the distant metastatic site was also identified as pivotal determinants of clinical outcomes, which was consistent with previous research [21]. Bone metastases may signify a more aggressive tumor phenotype and are potentially linked to dedifferentiation, which could render the cancer cells less manageable. Conversely, while lung metastases are frequent, they may imply a less invasive nature of the tumor cells. Additionally, lung metastases might exhibit a more favorable response to radioactive iodine therapy, whereas bone metastases generally elicit a suboptimal reaction to iodine treatments, thereby curtailing the therapeutic effect. The management of bone metastases through surgical intervention or other localized treatment approaches tends to be more complex, in contrast to lung metastases, which may benefit from a wider array of treatment alternatives. Furthermore, bone metastases can precipitate serious complications such as pathological fractures and hypercalcemia, adversely affecting patients' survival quality and prognosis.

Markers of inflammation have been increasingly recognized as significant factors associated with disease progression in DTC, particularly in cases complicated by distant metastasis [22, 23]. Our study suggests that a higher LMR correlates with a lower incidence of disease progression in DM-DTC patients. This finding emphasizes the role of the immune system in tumor behavior, whereby the activation of lymphocytes and monocytes is crucial for a robust immune response. Lymphocytes, particularly T cells, are integral to the adaptive immune response, providing targeted actions against tumor cells. Conversely, monocytes can differentiate into macrophages and contribute both to inflammatory processes and tissue remodeling. Hormonal and cellular mediators such as tumor necrosis factor α, interleukin-1, and interleukin-6 are key players in the early stages of inflammation, predominantly secreted by neutrophils, macrophages, and monocytes [24-26]. These cytokines can create a proinflammatory microenvironment that may facilitate tumor progression. The LMR serves as a simple, cost-effective marker for evaluating tumor-associated inflammation and has been associated with prognostic outcomes in various malignancies, including thyroid cancer [27-30]. A lower LMR is often indicative of a more aggressive tumor phenotype, characterized by sustained inflammation that aids in tumor growth and metastasis [31-33]. Thus, monitoring LMR alongside other inflammatory markers could enhance the prognostic assessment and therapeutic stratification of patients with DM-DTC. Further studies were warranted to clarify the underlying mechanisms and to determine whether modulation of the inflammatory response could become a therapeutic target in managing aggressive forms of thyroid cancer.

One of the primary limitations of this study was the relatively small sample size, which may not adequately represent the broader population of patients with DM-DTC. This limitation was compounded by the single-center retrospective design, which may introduce selection bias and limit the generalizability of the findings to other clinical settings or populations. Meanwhile, due to the limitation in sample size, the included independent variables did not encompass details of tumor burden, including the number of metastatic lesions, size of lesions, and other such metrics, which is one of the shortcomings of this study. Single measurements of peripheral blood parameters may be insufficient to represent the immune status during the progression of disease, and dynamic monitoring of marker changes may be necessary. This requires prospective studies for further validation.

In summary, this study highlights the prognostic value of inflammatory markers in predicting disease progression in patients with DM-DTC. Our findings suggest that LMR could serve as an important predictor for PD in DM-DTC patients. Inflammatory markers, combined with clinical-pathological features, can enhance the accuracy of prognostic models, potentially guiding personalized treatment strategies. Additionally, exploring the molecular mechanisms underlying the relationship between inflammatory markers and thyroid cancer progression could provide deeper insights and identify novel therapeutic targets.

## Data Availability

The data used to support the findings of the present study are available from the corresponding author on request.

## Abbreviations

CT: computed tomography
DM-DTC: distant metastasis differentiated thyroid cancer
FTC: follicular thyroid cancer
LMR: lymphocyte-to-monocyte ratio
NLR: neutrophil-to-lymphocyte ratio
PD: disease progression
PFS: progression-free survival
PLR: platelet-to-lymphocyte ratio
PTC: papillary thyroid carcinoma
RAIR: radioiodine-refractory
ROC: receiver operating characteristic
SII: systemic immune-inflammation index
sTg: stimulated thyroglobulin
Tg: thyroglobulin
TgAb: thyroglobulin antibodies
TSH: thyrotropin
